# A Mixed Methods Small Pilot Study to Describe the Effects of Upper Limb Training Using a Virtual Reality Gaming System in People with Chronic Stroke

**DOI:** 10.1155/2017/9569178

**Published:** 2017-01-18

**Authors:** Rachel C. Stockley, Deborah A. O'Connor, Phil Smith, Sylvia Moss, Lizzie Allsop, Wendy Edge

**Affiliations:** ^1^Department of Health Professions, Faculty of Health and Social Care, Manchester Metropolitan University, Manchester M15 6GX, UK; ^2^The Brain and Spinal Injuries Centre, Eccles Old Road, Salford, UK

## Abstract

*Introduction*. This small pilot study aimed to examine the feasibility of an upper limb rehabilitation system (the YouGrabber) in a community rehabilitation centre, qualitatively explore participant experiences, and describe changes after using it.* Methods and Material*. Chronic stroke participants attending a community rehabilitation centre in the UK were randomised to either a YouGrabber or a gym group and completed 18 training sessions over 12 weeks. The motor activity log, box and block, and fatigue severity score were administered by a blinded assessor before and after the intervention. Semistructured interviews were used to ascertain participants' views about using the YouGrabber.* Results*. Twelve participants (6 females) with chronic stroke were recruited. All adhered to the intervention. There were no adverse events, dropouts, or withdrawal. There were no significant differences between the YouGrabber and gym groups although there were significant within group improvements on the motor activity log (median change: 0.59, range: 0.2–1.25; *p* < 0.05) within the YouGrabber group. Participants reported that the YouGrabber was motivational but they expressed frustration with technical challenges.* Conclusions*. The YouGrabber appeared practical and may improve upper limb activities in people several months after stroke. Future work could examine cognition, cost effectiveness, and different training intensities.

## 1. Introduction

There are approximately 33 million stroke survivors worldwide [1]. Whilst the survival rate of stroke continues to improve, it is recognised that many survivors continue to be left with functional deficits that impact upon their quality of life and limit their return to vital functions and hobbies [1]. The ability to return to activities of daily living after stroke can be maximised by rehabilitative therapy which improves quality of life and facilitates independence [2]. A key component of physical therapy after stroke is repetition, or practice, of challenging movements that are focused on achieving a task or function [3]. This repeated task practice has been shown to facilitate and harness positive adaptations within the brain to aid recovery [4]. Whilst an ideal amount of practice to improve daily functioning has not been established [5], animal studies suggest that in excess of 400 repetitions are needed to promote plastic changes in the brain [3]. In clinical studies, two to three hours a day of practice for six weeks has been shown to elicit meaningful improvements in stroke survivors [6]. Meta-analyses of clinical trials also indicate that higher doses of practice promote better outcomes in impairments and activities of daily living for people after stroke [4, 7–9].

Facilitating increased practice of task orientated movements may be particularly helpful in improving the upper limb in people after stroke. Between half and two-thirds of stroke survivors report problems with their upper limb which significantly affects their activities of daily living [10] and has considerable negative effects upon participation and quality of life [11, 12]. Recovery of the upper limb may be particularly difficult as an individual's use of the affected arm has been observed to be minimal after stroke [13]. Furthermore, restoration of the upper limb often is not the primary aim of initial rehabilitation for both the patient and therapist, who are likely to be more focused on regaining the ability to walk [14]. Consequently it is unsurprising that less than 10 minutes of a typical therapy session are focused upon activities for the upper limb [13, 15].

There is good quality evidence for the use of interventions which require repetitive, task orientated, and task specific activities to improve the recovery of the upper limb after stroke [16, 17]. These interventions include constraint induced movement therapy (CIMT), virtual reality, and interactive video games [17]. Interactive video gaming using forms of virtual reality (VR) have grown in popularity as a method to increase repeated practice of challenging and engaging movements for people after stroke. Training using interactive VR games can provide task oriented, unpredictable and graduated learning [18], and augmented feedback regarding performance and results which motivate and engage players [19].

Although there is insufficient evidence to compare different upper limb interventions [16], the literature suggests that interactive VR game training is at least as effective as conventional exercises to elicit improvements in the upper limb after stroke [20]. However, many studies use a broad range of gaming systems in mostly acute stroke survivors where participants were typically based in hospital settings [20]. Consequently, the effects of virtual reality gaming upon the upper limb function of community dwelling stroke survivors who have had their strokes many months or years ago are not established. Furthermore, only a few studies have considered the views of participants about using virtual reality gaming systems for rehabilitation of the upper limb. Whilst these views have been largely positive, similar findings cannot be assumed between different training locations and gaming systems [21].

Therefore, this small, prospective mixed methods study was developed to

1. examine the feasibility of a custom made virtual reality upper limb interactive gaming tool called the YouGrabber® (YouRehab) in people after stroke who are attending a community outpatient rehabilitation centre,
2. describe the changes in upper limb function after using the YouGrabber and estimate the magnitude of the change in order to inform the sample size needed for a future trial,
3. explore the experiences of participants who had used the YouGrabber for rehabilitation of the upper limb after stroke.

## 2. Materials and Methods

A prospective, randomised, mixed methodology comprising feasibility assessment, qualitative semistructured interviews, and quantitative measurements was utilised.

Community dwelling stroke survivors who had a confirmed first stroke more than 6 months ago were prospectively recruited from a local rehabilitation centre (the Brain and Spinal Injuries Centre, Salford, UK) between October 2014 and May 2015. Participants were included if they complained of upper limb difficulties without pain, could move at least one block within 60 seconds on the box and block test, and were not severely cognitively impaired, scoring over 17 on the Montreal Cognitive Assessment [22–24].

Ethical approval was granted for the study by Manchester Metropolitan University Ethics Committee Reference Number: 1226. All participants gave informed consent.

*Intervention*. All participants completed baseline measurements of the primary outcome tool, the motor activity log (MAL) alongside secondary outcomes of the box and block test, and fatigue severity scale (FSS) which have been validated for people after stroke [22–26]. After baseline measurements, participants were randomly allocated by a blinded colleague using a computer based programme to either a YouGrabber (YG) or a gym group. The YouGrabber® (YouRehab) is a virtual reality system used in rehabilitation centres across Europe. It is a custom built interactive gaming system in which participants are seated in front of a screen and wear gloves containing sensors which relay real time information to a computer. The YouGrabber enables participants to play six games which focus upon dexterity (Airplane) mirror imaging (Magic Finger) and grasp and release in different positions (Toy Catching; Catch the Carrot; and Tomato Juggling). It gives visual, but not somatosensory, feedback upon attainment for each game.

The YG group completed 18 sessions of upper limb gaming therapy lasting approximately 30 minutes using the YouGrabber in addition to usual treatment which comprised therapeutic exercise in an onsite gym. In order to normalise for time and attention between groups, the control, gym group undertook personalised therapeutic exercise in the onsite gym with a longer training duration. The gym training for both YG and gym groups was individually prescribed by a physiotherapist but typically comprised power assisted exercise using machines which enable individuals to move through range passively, with assistance or against resistance depending upon their ability, stationary cycling, and stretches of large muscle groups.

Participants in both groups were asked to complete 18 sessions over 12 weeks with their training sessions recorded in a training diary by BASIC staff. The training frequency and duration were based upon the intensity of training used in other similar studies [20] and from a patient partner steering group who agreed that this was feasible for most people to complete. All participants received standby supervision from a physiotherapist whilst using the YouGrabber and in the gym.

The feasibility of the intervention was assessed by examining the ease and rate of recruitment to the study, willingness to be randomised, adherence, and withdrawal/dropout. Staff monitored and recorded any adverse events.

After completion of the 18 training sessions, the motor activity log with secondary outcomes of the box and block and fatigue severity scale were retested by a blinded assessor (PS).

Participants that had used the YouGrabber were interviewed by one researcher (DOC). Their carers/family members were also welcome to attend and contribute to interviews if they wished. The interview schedule elicited information about their perception of their upper limb problems, effects of the YouGrabber, benefits and challenges of using the device, duration, and frequency of use, and whether participants would wish to continue using it after the intervention ceased. Additionally participants were asked what changes (if any) they would like to make to the YouGrabber or the games used.

*Analysis*. Success of assessor blinding was determined by comparing the assessor guesses and actual group allocation after he completed the final assessments.

Differences in scores on the box and block, MAL, and FSS between the YG and gym groups were compared at baseline and changes in scores from baseline to the end of the intervention using Mann–Whitney *U* tests. Corrections for multiple comparisons were not made as [27]. Within group differences from baseline to completion of the training were analysed using Wilcoxon tests. *p* was set at <0.05 for all tests.

A simple sample size calculation for a future trial was performed using the primary outcome measure, mean MAL (amount), after the intervention with 80% power and *p* < 0.05 [28].

Interviews were recorded and transcribed verbatim. The data was analysed using a thematic approach. Recurring words or phrases were grouped and a process of familiarisation and simple coding was used for each interview question. Following this the common themes and subthemes were extracted and analysed to arrive at the final set of organising and then global themes [29]. Responses were read on a number of occasions to ensure clarity and accuracy of coding. A second researcher (RS) then verified coding for consistency and any differences were resolved by the use of a third, independent party.

## 3. Results

**Figure 1:**
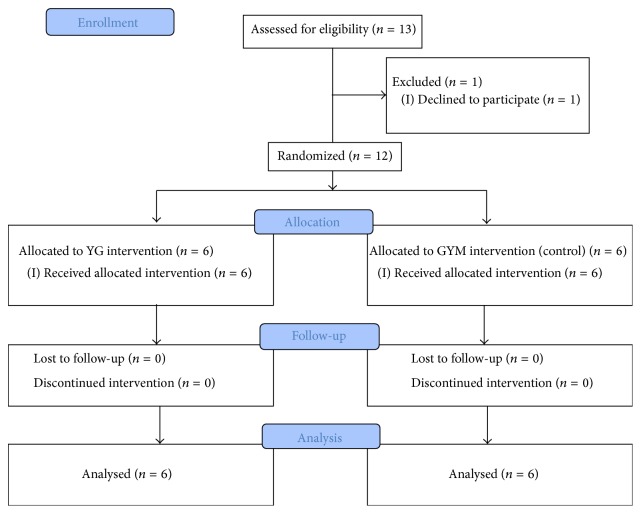
Consort flow diagram.

Recruitment began in October 2014 and ran until May 2015 (7 months). In this time, 13 (6 females) participants, a median of 2.2 years (range: 1.2–5.9), after stroke were approached to participate. This was the maximum number that was attending the centre for physical rehabilitation and met the inclusion criteria over the recruitment period. Twelve (6 females) consented and completed the trial (Figure 1). One person declined to participate. Ten participants were right handed; 3 had sustained an ischaemic stroke whilst 6 had sustained a haemorrhagic stroke. The cause of three participant's strokes could not be ascertained.

### 3.1. Feasibility

Eleven participants completed 18 training sessions in a median of 59 days (range 34–96); one participant (in the gym group) only completed 17 sessions. There were no adverse events and no participants were withdrawn or dropped out. Randomisation and blinding procedures were successful; no participant left the study after randomisation and the assessor only correctly guessed group allocation in 7 from 12 cases (58%).

There were no differences between groups in age (median age, years, range YG: 70.8; 58.5 to 82.3; gym: 70.6, 44.2 to 82) or in time since stroke (YG: 2.1 years, 1.2 to 5.9; gym: 2.2, 1.8 to 4.6).

Baseline participant details for each group are presented in Table 1.

### 3.2. Changes after the Intervention

#### 3.2.1. Within Group Changes after the Intervention

The median changes after the intervention as shown in Table 2 indicated small improvements in outcomes for both groups for all measurements except the fatigue severity scale which indicated a slight worsening of fatigue for both groups. These changes reached significance for both sections of the MAL and FSS for the YG group but were not significant for any measurement in the gym group.

#### 3.2.2. Between Group Differences

Despite somewhat larger changes in outcomes in the YG group, there were no significant differences between the YG and gym groups after completion of the programme (Table 2).

A sample size calculation using the primary outcome mean MAL (amount completed) scores after the intervention (YG: 2.00; gym: 1.73; common standard deviation: 1.52) for a power of 0.8 and a *p* value of 0.05 demonstrated that 495 participants would be needed in each group for a future controlled trial [28].

### 3.3. Participant Experiences

**Figure 2:**
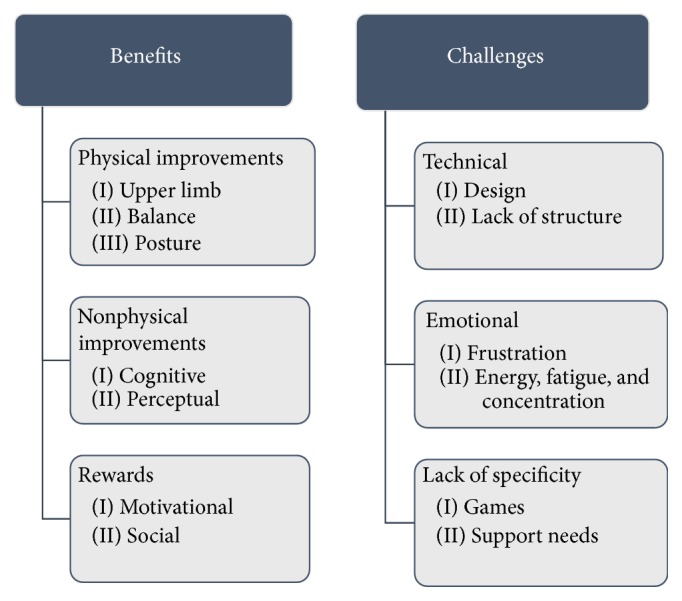
Themes from participant interviews.

Two global themes of benefits and challenges were established (as shown in Figure 2).

The main benefits derived from the interview data related to improvements and rewards. Specifically, participants highlighted a range of benefits in domains of physical functioning of the upper limb, balance, and gait, as well as cognitive, visual, and perceptual gains.

Rewards included motivation and achievement of goals, with several of the YG participants highlighting that they felt more confident completing a range of tasks after training.

“well… in terms of its ability to make my hand more mobile it certainly succeeded in that. I can now move all my fingers as you can see.” [Participant 1]

Participants' family members also observed improvements that the participants using the YouGrabber had not necessarily identified themselves.

“I think he's been more focused generally. So he's been less likely to shift his concentration and his eye movements and his thought process around.” [family member of Participant 2]

Participants also highlighted social rewards including increased participation, commitment to attending the centre for their rehabilitation, and feelings of increased control over their condition, upper limb, and mood.

“I enjoyed doing it because it's a bit of escapism. Because you're concentrating on that screen… I think initially it perhaps makes you lose yourself into something …and for that short period of time you're not thinking about what's happened to you.” [Participant 3]

“Yeah, I got quite a buzz out of it, yeah, that I was achieving highly.” [Participant 2]

Challenges included technological issues and design flaws such as freezing of the software and a lack of structure within the games on the YouGrabber device. There were notable emotional challenges including frustration with the machine and the game being played. Interviewees also reported frustration with themselves because sometimes their reduced ability meant that they were unable to complete the games on the YouGrabber.

“And it just froze, kept freezing basically, so I'd be moving my hand and nothing would be happening on the screen. Or the hand on the screen would be doing ridiculous movements that clearly wasn't me.” [Participant 4]

Participants reported within this theme that there were increased energy demands and increased concentration requirements which left them feeling tired. They also noted that they found it hard to pace themselves.

“It could be a little exhausting, you know. Strangely enough, you know sort of… get a little bit tired after it.” [Participant 3]

A further challenge related to a lack of specificity of the games provided by the YouGrabber. Participants felt that the six games were not sufficiently focused on specific issues such as dexterity and that some were too simplistic and not challenging.

“… the hand thing. It doesn't use the finger that I want it to use. It sort of does… you do a lot of this [demonstrates] and a lot of that [demonstrates] but you don't do anything with that one particular one. It seems to be omitted and I don't know why.” [Participant 5]

Several participants also highlighted a lack of structure in the progression of games and some felt that staff gave too much support whilst others wanted more assistance.

## 4. Discussion

This small pilot study set out to determine the feasibility of upper limb training using a custom designed virtual reality gaming device, the YouGrabber, to describe any changes in function and participants' experiences of using this gaming device.

The findings of this study showed that there were no adverse events when using the YouGrabber. Adherence to both the YouGrabber and gym programmes appeared excellent as all but one participant (in the gym group) completed 18 sessions in less than 9 weeks.

Whilst the rate of recruitment was somewhat slow (less than 2 participants a month) in comparison to others investigating upper limb treatments [30], the processes for randomisation and blinding were successful and so could be replicated in a future trial. However, substantial variability in MAL scores between participants means that a large (*n* = 990) number of participants would be needed in a future controlled trial. Whilst stratification based upon baseline upper limb activity level could reduce variability, a trial of this size would still likely require a multicentred, national or international design in order to recruit sufficient numbers in a reasonable timescale and would require significant funding.

The findings of this study should be considered in light of several limitations inherent to its design and structure. These include the relatively small sample and that all participants (in both YG and gym groups) were already attending the rehabilitation centre (BASIC). Whilst patients often report high quality, timely therapy interventions in the acute phase, the presence and content of continuing rehabilitation are variable which make a standardised control group comprising “usual care” unfeasible [31]. This also suggests that the participants in the current study maybe atypical of stroke survivors living in the community who may not, or choose not to, access ongoing rehabilitative services.

### 4.1. Comparison between YouGrabber and Gym Training

Participants in the YG group exhibited somewhat larger improvements in upper limb use in everyday activities measured by the MAL than the gym group, although this difference did not reach significance (*p* = 0.09). Neither group reached the minimally clinical important difference for the MAL in acute stroke survivors (>1.0) [32]. Whilst the study was not designed nor adequately powered to detect intergroup differences, it is possible that somewhat larger improvements within the YG group may have reached significance in comparison to the gym group with a greater training intensity. In their review, Thomson et al. (2014) found that participants could tolerate up to 180 minutes of upper limb training using computer based gaming per week without any adverse events [33]. This indicates that a greater training intensity than that utilised in the current study could be considered in a future trial of the YouGrabber to evaluate any differences to conventional rehabilitation. However, participants reported somewhat greater fatigue after the intervention (median change: YG: 0.67; gym: 0.75) on the FSS. This is echoed by participants' comments from the interview as they stated that the YouGrabber required increased energy demands and concentration which left them feeling tired. Whilst increased fatigue might be expected directly after completing a training session, the FSS asks participants about their fatigue in the preceding week [26]. These results agree with others who have reported slight increases in fatigue when using VR forms of rehabilitation [34]. The findings of somewhat increased fatigue after training suggests that it may be worsened further if a greater training intensity is used and that fatigue should be more closely monitored in future work. However, measurements of fatigue on the FSS were slightly larger in the gym group after training than the YouGrabber group suggesting that fatigue was not directly related to the YouGrabber intervention. It is perhaps more likely that both the YouGrabber and gym interventions slightly increased fatigue because of an increase in overall activity. Nonetheless, this finding does indicate that fatigue should be closely monitored in a future study.

The finding of increased upper limb usage in daily activities after using the YouGrabber is supported by others in people after stroke using differing forms of virtual reality gaming devices [35, 36]. It is also worth noting that, despite being a median average of 2.2 years after their stroke, participants still appeared to have the capacity to change and improve after the intervention. The ability to improve upper limb function months or even years after stroke is supported by reports from chronic stroke participants [7, 8] and suggests that therapists should consider instigating upper limb rehabilitation interventions irrespective of time since stroke.

#### 4.1.1. Neuroplastic Mechanisms

The neuroplastic mechanisms underlying changes in upper limb impairments after using virtual reality have not been fully elucidated. Training (45 minutes, 5 days/week, for 4 weeks) using the YouGrabber has been shown to produce increased initial activity in the bilateral sensorimotor cortex and supplementary motor areas measured using fMRI in two chronic stroke survivors [37]. With continued training, a shift in activity towards the affected hemisphere was observed [37] suggesting ongoing neuroplasticity despite the chronicity of the injury. In a larger study of 10 chronic stroke survivors, significant increases in sensorimotor activation, measured using electroencephalography, were seen after virtual reality based bilateral training when compared to 8 participants who completed bilateral upper limb training without virtual reality [38]. Whilst based on a small sample, this finding suggests that the presence of VR provided augmented stimulation which, in turn, produced greater remodelling of the brain compared to non-VR training. This lends support to the use of the YouGrabber or similarly challenging and repetitive VR interventions as part of rehabilitation after stroke.

### 4.2. Participant Views on Using the YouGrabber

Participants reported several benefits of using the YouGrabber. These included improvements in their reported usage of their upper limb but also perceived benefits to posture and balance. Bilateral arm training has shown some improvements in trunk control in other studies [39] most likely by improving control of the shoulder complex. However, few have considered posture when assessing VR based interventions. These findings suggest that these aspects should be measured in future work by inclusion of activity and/or impairment based measures such as the postural assessment scale and motion capture [40]. Cognitive and perceptual benefits were reported nonphysical adaptations after using the YouGrabber. These have not been reported in many stroke studies [20] but VR has been linked to moderate cognitive improvements in neurological populations [41]. This suggests that measures of cognition and perception should be included in future studies of the YouGrabber in order to capture any changes in these important aspects of functioning.

As shown in Figure 2, participants identified that the YouGrabber had several shortcomings. Technical challenges included problems with freezing and lack of specificity of the games, meaning that participants could become frustrated. These technical difficulties meant that standby supervision was needed on several occasions. This finding increases the demands on therapist time and could potentially limit the usefulness of the system in home/unsupervised settings. Participants also reported some challenges with their ability to play the games, stating that they would have preferred them to be progressive and specific to aspects of upper limb function. Many VR interventions provide direct, individualised feedback and require participants to improve performance to progress through levels which provides continual challenges and motivation [19]. However, participants reported that the YouGrabber games used in this study did not provide this information which meant that this important aspect of augmented feedback was absent. This could explain the lack of differences between the groups found in this study, as the lack of specificity and progression in the games meant that the challenges they provided were not markedly different to the gym programme completed by the control group. This challenges the efficacy of YouGrabber to continue to engage and motivate participants in the long term.

## 5. Conclusions

This small mixed methods pilot study aimed to examine the feasibility of the YouGrabber upper limb rehabilitation VR system in community dwelling chronic survivors and to describe and explore upper limb function and participant experiences after using the YouGrabber.

The results indicated that recruitment and randomisation procedures were feasible and there were no adverse events from training. However, based on the results of the current study, a future trial would require in excess of 850 participants, which would require a multicentred design.

There were several notable technical problems linked to the system which frustrated several participants and required increased supervisory input.

Both the gym and YG groups demonstrated some improvements after training. Whilst there were no significant differences between the groups, the YG group did exhibit some significant within group improvements in upper limb activities measured using the MAL, whilst the gym group did not. These findings suggest that training using the YG is at least as effective as gym based training and indicates that the stroke participants, who were at least over one year after stroke, could still exhibit significant benefits from training.

Participants using the YG reported that they experienced benefits in cognition and perception, but these aspects of functioning were not examined by the tools used in the current study. Small increases in fatigue were also evident both during participant interview and from questionnaire scores indicating that this aspect of functioning should be closely monitored in stroke patients undergoing rehabilitation. These findings highlight that a broader range of tools are needed in a future trial of the YouGrabber in order to capture aspects of cognition, perception, and fatigue. Future work should also examine the effect of training intensity of the YouGrabber, effects on trunk and posture, and its ability to be used with/without supervision and evaluate its cost effectiveness.

## Figures and Tables

**Table 1:** Baseline values for the YouGrabber and gym groups.

Parameter	Median	Range	Median	Range
Group	YouGrabber		Gym	
MAL Amt	1.2	0–3.4	1.1	0–2
MAL HW	1.3	0–2.5	1	0–2.7
Box and block	20.5	2–49	22.5	3–46
FSS	2.1	1–4.4	3.6	2–5.88

MAL: motor activity log, AMT: amount completed; a higher score indicates greater use, HW: how well; how well they perceived they used their upper limb; a higher score indicates greater use, and box and block: how many blocks moved in 60 seconds with affected arm. FSS: fatigue severity scale; a higher score indicates greater fatigue.

**Table 2:** Median changes after completion of the intervention.

Parameter	Final values (range)	Median change from baseline (range)	Within group *p* value	Final values (range)	Median change from baseline (range)	Within group *p* value	Between group *p* value
Group	YouGrabber			Gym			
MAL Amt	1.65 (0.4–4.6)	0.59^*∗*^ (0.2–1.25)	0.03	1.4 (0.1–4.1)	0.13 (−0.23–3.1)	0.17	0.13
MAL HW	1.75 (0.47–3.3)	0.56^*∗*^ (0.27–0.35)	0.03	1.6 (0.1–3.1)	0.09 (0.46–2.17)	0.35	0.09
Box and block	22.5 (6–75)	3 (−3–26)	0.25	23.5 (2–49)	0.5 (−5–7)	0.6	0.49
FSS	4.1 (1.34–5.9)	0.67^*∗*^ (0.3–2.78)	0.03	4.7 (2.9–6.3)	0.75 (−0.77–2.7)	0.29	0.59

*∗* denotes a significant difference within groups; the *p* value denotes within group significance level. MAL: motor activity log, AMT: amount completed; how much they used their upper limb; a higher score indicates greater use, HW: how well; how well they perceived they used their upper limb; a higher score indicates greater use, and box and block: how many blocks moved in 60 seconds with affected arm. FSS: fatigue severity scale; a positive score indicates increased fatigue.
